# Genome-wide association analysis for drought tolerance and associated traits in faba bean (*Vicia faba* L.)

**DOI:** 10.3389/fpls.2023.1091875

**Published:** 2023-02-01

**Authors:** Natalia Gutiérrez, Marie Pégard, Christiane Balko, Ana M. Torres

**Affiliations:** ^1^ Área de Mejora y Biotecnología, Andalusian Institute of Agricultural and Fisheries Research and Training (IFAPA), Centro Alameda del Obispo, Córdoba, Spain; ^2^ INRAE P3F, 86600 Lusignan, France, INRA, Centre Nouvelle-Aquitaine-Poitiers, Lusignan, France; ^3^ Julius Kühn-Institut (JKI), Federal Research Centre for Cultivated Plants, Institute for Resistance Research and Stress Tolerance, Sanitz, Germany

**Keywords:** drought stress, GWAS, genetic diversity, faba bean, PCA, heritability, SNPs markers, candidate genes

## Abstract

Faba bean (*Vicia faba* L.) is an important high protein legume adapted to diverse climatic conditions with multiple benefits for the overall sustainability of the cropping systems. Plant-based protein demand is being expanded and faba bean is a good candidate to cover this need. However, the crop is very sensitive to abiotic stresses, especially drought, which severely affects faba bean yield and development worldwide. Therefore, identifying genes associated with drought stress tolerance is a major challenge in faba bean breeding. Although the faba bean response to drought stress has been widely studied, the molecular approaches to improve drought tolerance in this crop are still limited. Here we built on recent genomic advances such as the development of the first high-density SNP genotyping array, to conduct a genome-wide association study (GWAS) using thousands of genetic polymorphisms throughout the entire faba bean genome. A worldwide collection of 100 faba bean accessions was grown under control and drought conditions and 10 morphological, phenological and physiological traits were evaluated to identify single nucleotide polymorphism (SNP) markers associated with drought tolerance. We identified 29 SNP markers significantly correlated with these traits under drought stress conditions. The flanking sequences were blasted to the *Medicago truncatula* reference genomes in order to annotate potential candidate genes underlying the causal variants. Three of the SNPs for chlorophyll content after the stress, correspond to uncharacterized proteins indicating the presence of novel genes associated with drought tolerance in faba bean. The significance of stress-inducible signal transducers provides valuable information on the possible mechanisms underlying the faba bean response to drought stress, thus providing a foundation for future marker-assisted breeding in the crop.

## Introduction

Grain legumes are among the most important sources of high-protein for food and feed worldwide and represent key crops for sustainable, low-input, and diverse farming systems. In crop rotations, legumes enhance soil fertility through biological nitrogen fixation and break disease cycles, thus reducing the input of chemicals in agriculture (Nemecek et al., 2008). With one of the highest protein contents and a balanced amino acid profile, faba bean (*V. faba* L.) is the sixth global temperate legume in production (5.7 Million tonnes in 2.7 Mhas), after, chickpea, pea and lentil, with the average yield largely surpassing all of these crops (FAOSTAT, 2020). Faba bean can adapt to a variety of climatic and soil conditions, providing an advantage over other legume crops. Despite these benefits, faba beans still have a limited use in modern agriculture, mainly due to yield instability caused by biotic and abiotic (mainly drought) stresses. In the Mediterranean region, grain legumes are typically grown in rainfed agricultural systems and therefore yield is often variable or low due to the terminal droughts that characterise these areas (Amede et al., 2003; Khan et al., 2007). As a result of climate change, droughts are predicted to increase both in frequency and intensity, further hampering acceptance and wider use of faba beans by farmers in this area as well as in Northern Europe.

Its high sensitivity to drought stress from seedling to maturity (Khan et al., 2007; Khan et al., 2010) prevents faba bean from expressing its full performance potential. A meta-analysis synthesizing the results of field studies and drought experiments across the globe along 34 years revealed a reduction of 40% in faba bean yield following a 65% decrease in water availability, the highest drought-induced yield reduction among the surveyed legume crops (Daryanto et al., 2015). Thus, identifying drought-tolerant faba bean genotypes and developing efficient molecular breeding approaches is crucial to mitigate the devastating impact of drought stress.

A wide genetic variation has been reported in faba bean accessions for various traits related to drought adaptation (Muktadir et al., 2020). In other legume crops, selection for drought resistance based on highly heritable secondary traits, together with physiological attributes such as accumulation of proline or soluble sugars, has proven highly successful (Lafitte et al., 2003; Richards, 2006; Stoddard et al., 2006; Annicchiarico and Iannucci, 2008; Alderfasi and Alghamdi, 2010; Ammar et al., 2015; Balko et al., 2023, submitted). Although the faba bean response to drought stress has been widely studied (Ricciardi et al., 2001; Ammar et al., 2015; Siddiqui et al., 2015), few molecular approaches have been taken to improve drought tolerance in this crop. Khazaei et al. (2014) first reported quantitative trait loci (QTLs) for stomatal characteristics located on chromosome II and exploited the synteny with the model legume species *M. truncatula* to identify candidate genes within these QTLs. Subsequently, Abid et al. (2015) identified six putative drought stress responsive genes in faba bean by suppression subtractive hybridization. More recently, Ali et al. (2016) published the first and so far only drought genome-wide association analysis (GWAS) focusing on a set of 189 German winter faba bean lines derived from 11 parental founders, by assessing a number of physiological aspects related with dehydration tolerance established in previous studies (Balko et al., 1995; Lafitte et al., 2003; Balko, 2005; Stoddard et al., 2006; Richards, 2006; Annicchiarico and Iannucci, 2008; Alderfasi and Alghamdi, 2010; Ammar et al., 2015; Balko et al., 2023, submitted). Using 175 single-nucleotide polymorphisms (SNPs) and 1147 amplified fragment length polymorphisms (AFLPs), several QTLs were detected but the relatively low number of markers used and the very low linkage disequilibrium (LD) detected, limited the success of this association analysis.

In general, traits that contribute to drought tolerance in plants are quantitative and involve multiple genes. Therefore, it is crucial to exploit new genomic resources for the improvement of this crop. Recent advances in next-generation sequencing (NGS) and high-throughput genotyping have allowed the development of new faba bean genomic tools and resources, including mining of SNPs from transcriptome data (Kaur et al., 2014; Ocaña et al., 2015; Webb et al., 2016) and the development of the first high-density SNP genotyping array (O’Sullivan et al., 2019). These resources allow us to conduct genome-wide association studies (GWAS) using thousands of genetic polymorphisms distributed throughout the entire genome. GWAS aims at identifying genetic markers that are strongly associated with QTLs by using the LD between the genetic marker and the causal mutation. Compared with linkage mapping, GWAS provides higher allelic diversity at the corresponding loci and exploits ancestral recombination events in a population, leading to a better association between the marker and the phenotype of a target trait (Zhu et al., 2008).

In recent years, GWAS studies have been conducted in many plant species to dissect complex quantitative traits related to drought tolerance (Hoyos-Villegas et al., 2017; Dossa et al., 2019; Dramadri et al., 2021; Ravelombola et al., 2021; Choudhary et al., 2022). As stated above, a single GWAS study on drought tolerance has been conducted so far in faba bean (Ali et al., 2016), whose results were limited by the low LD and number of markers. The objectives of the present study were: (1) to evaluate the drought tolerance index in faba bean of ten morphological, phenological and physiological traits, (2) to conduct GWAS to identify SNP markers associated with the drought tolerance indices; (3) to investigate the potential relationship between significant loci associated with the drought tolerance indices.

## Materials and methods

### Plant material

A panel of 100 faba bean accessions from different countries in Africa (8 accessions), North and South America (2), Asia (27) and Europe (39) were used in the study. The original country of the remaining 24 ICARDA accessions is unknown. Europe with 9 countries is the most represented geographical area in the panel, followed by Asia, Africa and America (7, 4 and 2 countries, respectively). Spain is the country accounting for the highest number of accessions (23). The panel includes genetic stocks, landraces and breeding lines aiming at gathering a wide range of genetic diversity from diverse geographical origins. The drought panel was made in collaboration with four public institutes: ICARDA, IFAPA, INIA and INRA, holding the following genebanks SYR002, ESP046, ESP004, FRA043, respectively. Prior to the genotyping analysis, all the Spanish lines had been selfed for at least four generations. The remaining accessions were purified for two generations by single seed descent (SSD) in insect-proof cages. A detailed description of the collection is provided in **Supplementary Table 1**.

### Phenotypic data analysis

#### Phenotyping

The 100 faba bean panel was assayed in 2019 and 2020 at the Julius Kühn-Institut (JKI) in Groß Lüsewitz, Germany (54.0701 N 12.33874 E), in a slightly loamy sand soil with pH: 5,7 (Balko et al., 2023 submitted). Field management essentially followed normal local faba bean cropping practices. Plants were sown in a randomized block design with four replications under irrigated (control) and drought stress conditions created under rain-out shelters (two blocks in each shelter). The accessions were grown in single row plots of 1.2 m length with 14 plants each and a row-to-row distance of 0.5 m. Drip irrigation was scheduled in the range of 60 - 70% of field capacity of the soil, determined over winter after excessive rainfall (Balko et al., 2023, submitted). Water content in the soil was assessed by Time Domain Reflectometry (TDR) probes in about 40 cm depth. Drought stress was initiated when about 30% of the plots started flowering. Irrigation in the stress treatment was stopped and during occurring rainfall the shelters were moved over the respective plots.

Six morphological and phenological traits were recorded: maturity date (MAT), defined as the date when more than 90% of the pods have ripened; plant height in cm (PH); number of pods per plant (PP); number of seeds per plant (SP); hundred seed weight (HSW) in grams; and plot yield (PY) in kg. Moreover, four physiological traits were assessed in leaves: free proline content (PRO) (Bates et al., 1973); total content of soluble sugars (TSS) (Yemm and Willis, 1954); and chlorophyll content (SPAD1 and SPAD2). SPAD measurements were performed with a Chlorophyll Meter SPAD 502 plus (Konica Minolta) at the beginning of the stress treatment (SPAD1) and 4 weeks after the onset of stress (SPAD2). Leaf samples for determination of free proline and total content of soluble sugars were taken in the same time window and flash frozen in liquid nitrogen (Balko et al., 2023 submitted). The measurements of these traits were performed by selecting ten individual plants in the middle of the row for each accession.

#### Adjustment of phenotypic data

All phenotypic traits were independently adjusted for field micro-enviromental heterogeneity using the ‘breedR’ package (Muñoz and Sanchez, 2022). Phenotypes were combined and adjusted by years. In the model, the genomic estimated breeding values (GEBV) for each trait were determined with the genomic best linear unbiased prediction based model (GBLUP) (Whittaker et al., 2000; Meuwissen et al., 2001; Cantet et al., 2005). Within trials, a random effect was fitted thanks to the use of the tensor product of two bi-splines bases with a covariance structure for the random knot effects (RKE) to account for spatial variability along the row (r) and the column (c) of the field design (Cantet et al., 2005; Cappa and Cantet, 2007; Cappa et al., 2015) to capture the spatial heterogeneity at the plot level. The following model was used:


y=μ+Zu+Ws+ϵ

where *y* is the raw phenotype, *µ* the global mean, *u* the vector of random additive effects following N(0,Gσ^2^_a_) with σ^2^_a_ the additive variance and G the relationship matrix, s the vector of random spatial effects containing the parameters of the B-splines tensor product following N(0,Sσ^2^_s_) with σ^2^_s_ the variance of the RKE for row and column and S the covariance structure in two dimension, ϵ the vector of residual effects following N(0,I σ^2^_e_) with σ^2^_e_ the residual variance. The design matrix *Z*, and *W* are indicator matrices relating the plot to the random effects. The method used to obtain the relationship matrix is detailed in the following section. Bi-splines were anchored at a given number of knots for rows and columns, a higher number of knots smooths out the surfaces. ‘breedR’ optimized the knot numbers by an automated grid search based on the Akaike Information Criterion (AIC). The micro-environmental individual effect was subtracted from the observed phenotype to obtain a spatially adjusted individual phenotype. A genotypic mean of the spatially adjusted phenotypes was calculated for each trait and used for the GWAS. All measurements were tested for deviations from normality by a randomized Q-Q plot.

### Genomic data analysis

#### Genotyping

Young leaves from individual plants were collected, ground in liquid nitrogen and total genomic DNA was isolated using the DNeasy Plant Mini Kit (QIAGEN Ltd, UK). DNA quality was checked by agarose gel electrophoresis and concentration was estimated using the QubitTM dsDNA BR Assay Kit (Invitrogen by ThermoFisher Scientific, UK) following the manufacturer’s instructions.

For genotyping we used the Vfaba_v2 Axiom SNP array with 50K SNP (O’Sullivan et al., 2019; Khazaei et al., 2021). Seven of the 100 accessions showing poor DNA quality, as well as SNP markers with a call rate below 97% and a minor allele frequency (MAF) below 95% were excluded from the analysis. After quality control, a matrix consisting of 21,915 SNPs and 93 accessions with 0.89% missing data was kept for further GWAS analysis. The imputation of the missing data was performed by mean allelic frequency. To obtain the genetic position of the significant SNPs we used the information and protocols provided by Skovbjerg et al., 2022. Thus, 17,403 out of the 21,915 SNPs markers could be assigned to genomic positions. The extremely large size of chromosome 1 (> 3 Gbp) generated problems with various softwares and therefore the chromosome was split at the centromere to form Chr1S and Chr1L. In addition, to verify and complete the faba bean chromosomal positions, the SNPs flanking sequences were aligned to the *V. faba* reference genomee^1^ (Jayakodi et al., 2022) using the Geneious v.7.1.9genomee^2^.

#### Genomic relationship matrix

The genomic relationship matrix (GRM) was constructed based on VanRaden, 2008, where the matrix Z was calculated as (M - P). M is a matrix of minor allele counts (0, 1, 2 for the reference, heterozygote and alternative, respectively) with *m* column (one for each marker) and *n* rows (one for each accession). P is a matrix which contains the allele frequency, expressed as a difference from 0.5 and multiplied by 2, such that column *i* of P is 2(p*_i_*-0.5). Subtraction of P from M gives Z, which sets mean values of the allele effects to 0. Genomic relationship matrix G was obtained for the first method proposed by VanRaden:


$$G=ZZʹ/2\sum^{\;}pi\left(1-pi\right)$$

#### Genetic structure

To estimate the number of distinct genetic clusters (K) and admixture existing in the faba bean panel a bayesian based clustering analysis was performed using FastSTRUCTURE v 1.0 (Raj et al., 2014). FastSTRUCTURE was run on default settings with 10-fold cross validation on the 100 accessions testing for subpopulations (K) values ranging from 1 to 10. The most likely K number was chosen by plotting the marginal likelihood of each model as a function of K and determining when the graph begins to plateau. Accessions with membership probabilities ≥ 0.50 were considered to belong to the same group. The choice of K was further supported by applying a discriminant analysis of principal components (DAPC) based procedure for clustering using the ‘fviz_pca’ function in the ‘factoextra’ R-package (Kassambara and Mundt, 2020).

### Correlation and broad sense heritability estimates

To understand the extent of the relationship among the traits, the correlation matrix for control and stress values was made by Pearson correlation analysis. Descriptive analysis and correlations were conducted in the R statistical software. The broad sense heritability (***h^2^***) for the traits was estimated using the following formula:


h2 = Vg/(Vg+Vsp+Vres)

where ***V_g_*** is the genetic variance component, ***V_sp_*** is the spatial variance component, ***V_res_*** is the residual variance component. The genotypic mean value for each accession for each trait under control and stress conditions were represented by mean PCA biplot. PCA was performed in the R software package ‘prcomp’ and visualized with the ‘fviz_pca’ function.

### Genome-wide association analysis

Association analyses were performed in 93 faba bean accessions with 21,915 high-quality SNPs. A Multi Locus Mixed Model method (MLMM) (Segura et al., 2012) was implemented in the R package ‘mlmm.gwas’ (Bonnafous et al., 2019) to evaluate the trait-SNP associations. The MLMM, is an iterative approach that improves power over single locus methods by incorporating multiple markers in the model simultaneously as covariates, to reduce the false-positive rates and to increase the detection power. In each step (maximum 10 steps), the variance components are estimated and then used to calculate p-values for the association of each SNP with the trait of interest. MLMM utilizes eBIC (extended Bayesian Information Criterion) to determine the number of steps and therefore the number of QTLs with a lambda value of 0.77. The Bonferroni threshold was used to label an association as significant. Significant markers were visualized with a Manhattan plot and important p-value distributions (expected vs. observed p-values on a -log10 scale) were shown with a quantile–quantile (Q-Q) plot.

### Potential candidate gene

The sequences flanking associated SNPs were blasted against the NCBI *M. truncatula* reference genome^3^ to annotate potential candidate genes underlying the causal variants. Gene locations were determined using the Genome Data Viewer (GDV)^4^. In addition, the sequences flanking associated SNPs were blasted against the faba bean reference genome (Jayakodi et al., 2022) to verify chromosomal positions and locate those candidates that did not show significant hits in *Medicago*. For some of these, the use of the corresponding faba bean contig allowed to infer the Medicago ortholog and include the corresponding gene annotation.

## Results

### Phenotypic variation and heritability

The ten morphological, phenological and physiological traits listed above were used to examine the possible existence of significant phenotypic variances among the 93 faba bean accessions, both in control and drought conditions. Descriptive statistics revealed large phenotypic variations for all the traits studied (**Table 1**). For MAT, PH, PP, SP, HSW, PY and SPAD2 the mean values in the drought stress treatment were lower than in the control condition. In contrast, PRO, TSS highly increased under drought stress while in SPAD1 the mean increase was smaller.

**Table 1:** Statistical analysis of 10 morphological, phenological and physiological traits in controlled and drought stress conditions.

Traits	Description	Treatment	Mean	Min	Max	Range	SD	CV (%)	*h^2^*
**MAT**	Maturity date (days)	Control	127.54	114.00	148.00	34.00	6.85	5.37	0.51
		Stress	115.08	101.00	132.00	31.00	5.93	5.16	0.52
**PH**	Plant height/cm	Control	64.59	22.67	127.60	104.93	15.35	23.76	0.56
		Stress	56.63	31.90	84.50	52.60	8.21	14.50	0.52
**PP**	Number of pods per plant	Control	10.84	2.63	36.25	33.62	5.26	48.53	0.61
		Stress	6.41	1.88	15.00	13.13	2.25	35.05	0.62
**SP**	Number of seeds per plant	Control	24.90	3.75	107.10	103.35	14.20	57.01	0.62
		Stress	14.20	3.13	36.20	33.08	5.44	38.27	0.57
**HSW**	100 seed weight/grams	Control	67.85	19.59	144.23	124.64	22.62	33.34	0.66
		Stress	60.88	17.22	123.46	106.24	16.47	27.06	0.78
**PY**	Plot yield/kg	Control	0.16	0.02	0.44	0.42	0.07	46.75	0.42
		Stress	0.08	0.02	0.24	0.23	0.03	38.71	0.32
**PRO**	Free proline content/μmol g−1	Control	2.22	0.71	14.75	14.04	0.85	38.10	0.30
		Stress	6.40	1.00	73.99	72.99	8.31	28.37	0.21
**TSS**	Total content of soluble sugars/μmol g−1	Control	1119.91	332.70	2602.00	2269.30	336.37	30.36	0.28
		Stress	1391.26	523.00	2886.95	2363.95	394.73	28.37	0.53
**SPAD1**	Chlorophyll content, beginning of stress	Control	37.62	24.80	48.00	23.20	4.05	10.77	0.75
		Stress	39.40	24.80	50.60	25.80	3.97	10.09	0.71
**SPAD2**	Chlorophyll content, 4 weeks after stress	Control	42.79	13.50	61.60	48.10	7.14	16.69	0.62
		Stress	23.70	9.40	56.40	47.00	8.61	36.33	0.69

**Figure 1:**
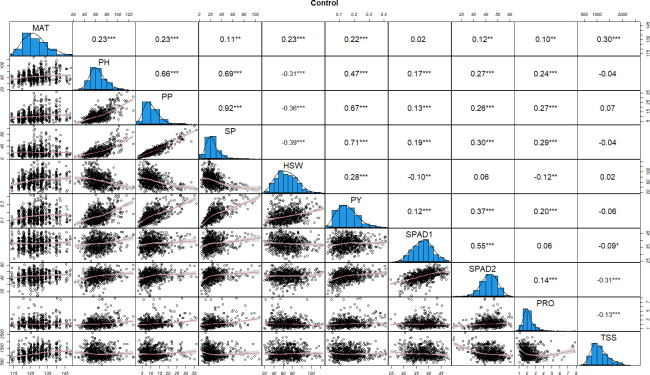
Distributions of phenotypic frequency and correlations between 10 morphological, phenological and physiological traits in control conditions. The frequency distribution of each trait is shown on a central diagonal in the form of a histogram. Scatter plots of correlations between every pair of traits are shown in the areas below the diagonal, and numerical Pearson’s correlation coefficients (r), between every pair of traits are shown in the areas above the diagonal. The red line in the scatter plots represents the slope of the correlations. The x- and y- axes are the values of the measurements (PH in cm, HSW in grams and PY in kg). *, ** and *** indicate significance at P < 0.05, P < 0.01 and P < 0.001, respectively.

The frequency distributions of all 10 traits fit the normal distributions, indicating their quantitative nature (**Figure 1**). The coefficient of variation (CV%) for most of the traits was comparable for control and drought stress. CV ranged from 5.37 (MAT) to 57.01 (SP) under control condition and from 5.16 (MAT) to 38.71 (PY) under drought stress. Narrow-sense heritability (*h^2^*) estimates ranged from 0.28 (TSS) to 0.75 (SPAD1) in the control and from 0.21 (PRO) to 0.78 (HSW) in drought stress. Heritabilities calculated for each trait were moderate to high for most of the traits, varying from 0.51 to 0.75 in control conditions and from 0.52 to 0.78 in drought stress. Slightly lower values were recorded for PY and PRO in both conditions. Except for TSS and in both treatments, similar estimates for heritability were detected. Under drought stress a lower coefficient of variation was observed in most traits with exception of (**Table 1**).

### Correlation of traits

To understand the relationship among the traits, we performed a correlation matrix for control and stress values using the Pearson correlation method. Under control conditions (**Figure 1**), significant positive correlations were observed among most of the traits, with correlation coefficient (r) values ranging from 0.10 to 0.92. By contrast, SPAD1 and PRO were negatively correlated with TSS (-0.09, -0.13, respectively), HSW was negatively correlated with SPAD1 and PRO (-0.10, -0.12, respectively) and PH, PP and SP with HSW (-0.31, -0.36 and -0.39, respectively). TSS revealed a close to neutral correlation with most of the physiological, morphological and phenological traits except MAT (0.30) and SPAD2 (-0.31), where highly significant positive and negative associations, respectively, were observed (**Figure 1**).

**Figure 2:**
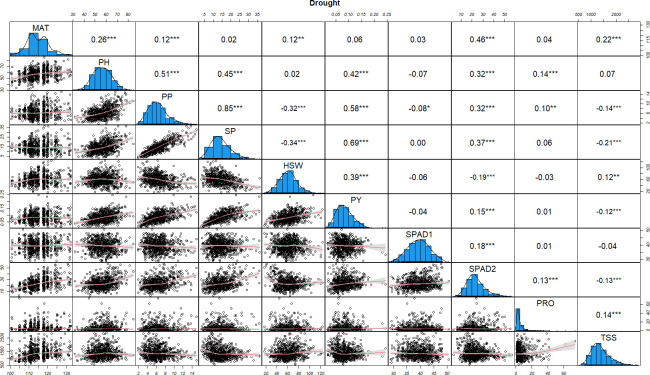
Distributions of phenotypic frequency and correlations between 10 morphological, phenological and physiological traits in drought stress conditions. The frequency distribution of each trait is shown on a central diagonal in the form of a histogram. Scatter plots of correlations between every pair of traits are shown in the areas below the diagonal, and numerical Pearson’s correlation coefficients (r), between every pair of traits are shown in the areas above the diagonal. The red line in the scatter plots represents the slope of the correlations. The x- and y- axes are the values of the measurements (PH in cm, HSW in grams and PY in kg). *, ** and *** indicate significance at P < 0.05, P < 0.01 and P < 0.001, respectively.

Similarly, under drought stress conditions most of the phenological and yield related traits (MAT, PH, PP, SP, HSW and PY), were strongly associated, showing positive correlations with Pearson’s correlation coefficients ranging between 0.12 and 0.85 (**Figure 2**). PP and SP showed a significant negative correlation with HSW (-0.32 and -0.34, respectively). SPAD1 maintained a neutral or significantly negative correlation with all the traits while SPAD2 was positively correlated with all of the characters except with HSW (-0.19). SPAD1, PRO and TSS showed negative or close to neutral correlation among them and with the rest of yield related traits. The only exceptions were the significant positive correlation of PRO with PH, PP, SPAD2 and TSS (0.14, 0.10, 0.13, 0.14), and the positive association of TSS with MAT (0.22).

Correlations in control and drought treatments provide useful information on the effect of the physiological parameters (SPAD, PRO and TSS) on the yield related traits studied. In both conditions, SPAD2 (the main indicator for drought stress induced leaf senescence), was significantly correlated with PP, SP and PY, with higher correlations observed under drought stress with PP and SP (0.32 and 0.37, respectively). Lower, but still significant correlations were also detected between PP, SP and PY with PRO in control conditions, while under drought stress only a slight correlation between PP and PRO (0.10) was detected. TSS was not significantly correlated with any of the yield related traits in control conditions whereas in stress conditions, highly significant negative correlations with PP, SP and PY (-0.14, -0.21 and -0.12) were observed.

### Genetic structure

**Figure 3:**
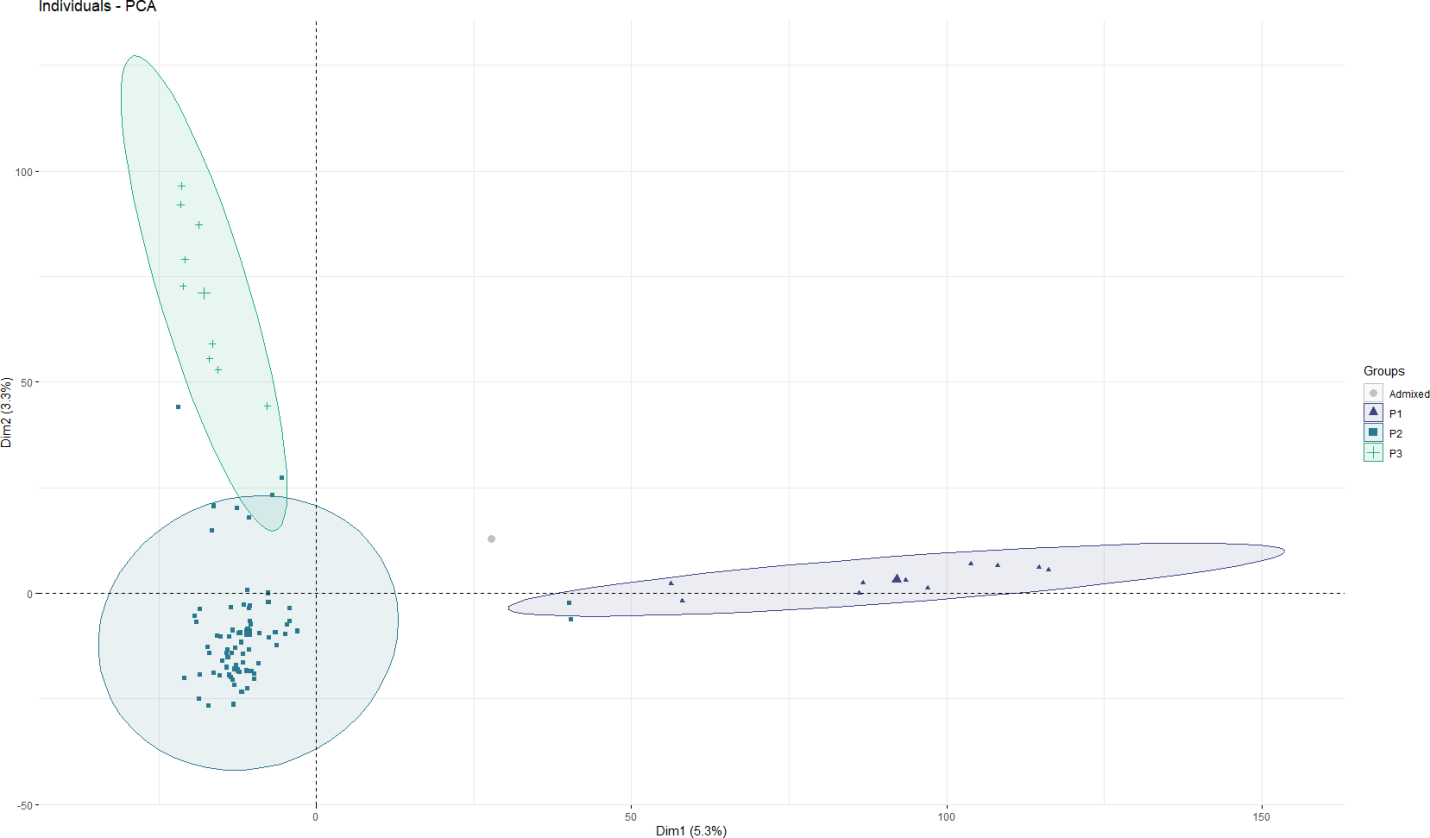
Principal component analysis (PCA) of the 93 faba bean accessions. Each dot represents an accession. The horizontal and vertical coordinates represent the first two principal components of analysis (PC1 and PC2), accounting for 5.3% and 3.3% of the total variation, respectively.

To examine divergence of the faba bean collection during evolution, a Bayesian based clustering analysis was performed using FastSTRUCTURE and the 21,915 selected SNPs. According to the K genetic clusters, the most likely number of inferred members was three with K ≥ 0.50. Besides, we performed PCA using the first two principal components, PC1 (variance explained, 5.3%) and PC2 (variance explained, 3.3%), which are divided into three groups with slight degrees of introgression between them during cultivation (**Figure 3**). Clade P1 comprised only oriental accessions (10) from China, Nepal and Japan. Clade P2 was the most numerous and consisted of 73 accessions with a wide range of geographic origins spread over four continents: Europe (25), Africa (7), Asia (18) and South America (1), while the remaining 22 accessions are of unknown origin. The third clade (P3) mainly consisted of European accessions (7) together with one from Canada and another from Egypt. Accession EUC_VF_192 was admixed (**Supplementary Table 1**).

### Principal component analysis biplot

**Figure 4:**
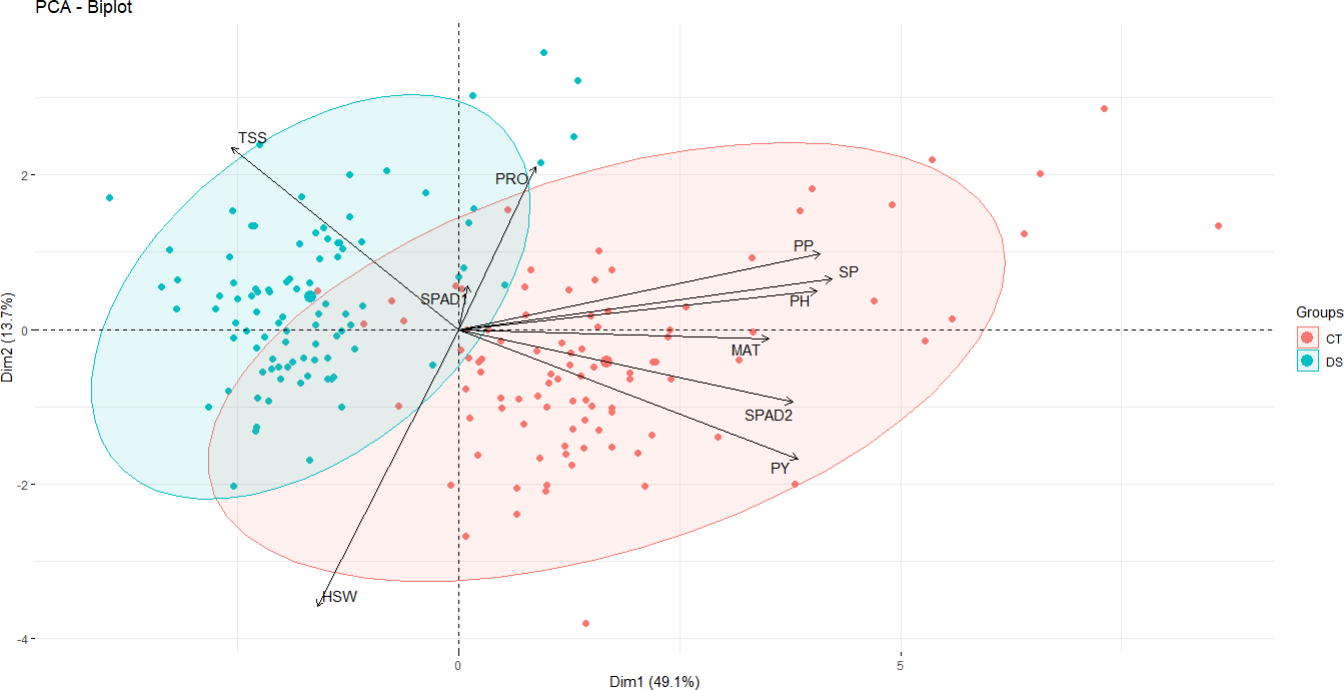
PCA biplot showing the clustering of 93 faba bean accessions grown under control and drought stress conditions based on the variance in 10 morpho-physiological and biochemical traits. The traits are maturity date (MAT), plant height (PH), number of pods per plant (PP), number of seeds per plant (SP), 100 seed weight (HSW) and plot yield (PY), free proline content (PRO), total content of soluble sugars (TSS) chlorophyll content (SPAD1 and SPAD2). The first two components explained 49.3% and 18% of the variances, respectively. The magnitude of the vectors (arrows) shows the strength of their contribution to each PC. Vectors pointing in similar directions indicate positively correlated variables, vectors pointing in opposite directions indicate negatively correlated variables, and vectors at proximately right angles indicate low or no correlation. Colored concentration ellipses (size determined by a 0.95-probability level) show the observations grouped by treatment (control or drought conditions). Individuals on the same side as a given variable should be interpreted as having a high contribution on it.

The contribution of the various traits to the overall variation in the dataset was investigated by PCA (**Figure 4**). The first three principal components (PCs) with eigenvalues > 1 accounted for 74.4% of the total variation (**Supplementary Figure 1**). Since the first two PCs showed the highest percentage of variance (62.8%), the PCA biplot was constructed only with PC1 and PC2, showing a clear separation of control vs. drought stress data points along the main axis (**Figure 4**). PC1 explained 49.1% of the total variability among traits or individuals and was mostly associated with SP, PP, PH, PY, SPAD2 and MAT (**Figure 4**). PC2 accounted for an additional 13.7% of the total variability among traits and appeared to be related with HSW PRO and TSS (**Figure 3**). PC3, PC4 and PC5 explained only 11.66%, 9.5% and 7.6%, respectively, of the phenotypic variation (**Supplementary Figure 1**). PC2 was highly associated with HSW, PRO and TSS. PC3 was strongly associated with SPAD1 and PC with PRO (**Supplementary Figure 2**). The biplot vectors showed that the morphological, phenological and yield-related traits (PP, SP, PH, PY, MAT) show a strong positive correlation between each other and with the physiological trait SPAD2, while HSW has a less strong correlation and instead shows a strong negative correlation with PRO and SPAD1 (**Figure 4**).

### Genome-wide association mapping

**Figure 5:**
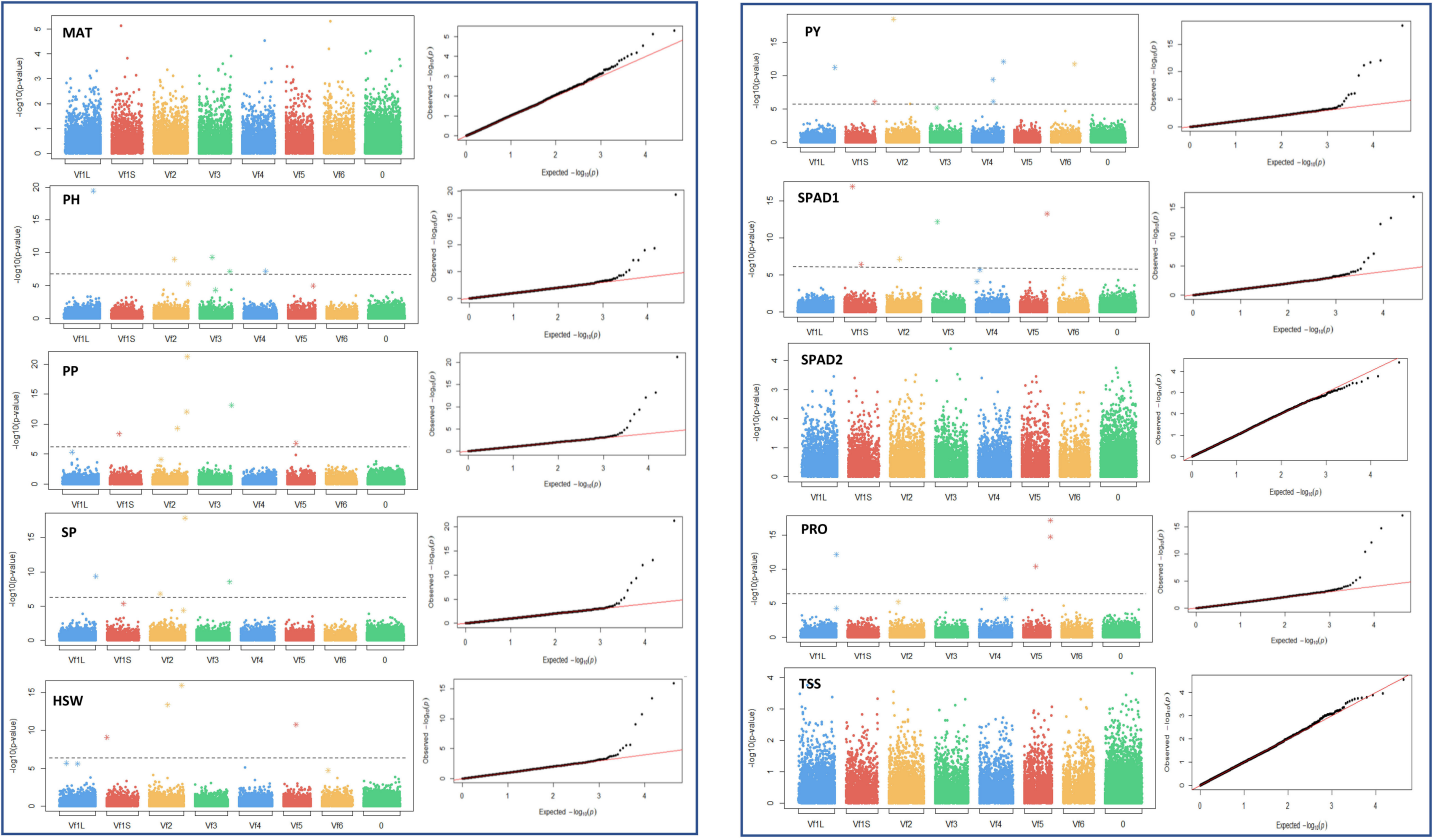
Manhattan plots and quantile-quantile (Q-Q) plots of the GWAS results for the 10 traits studied in control conditions. MAT, maturity date; PH, plant height; PP, number of pods per plant; SP, number of seeds per plant; HSW, 100 seed weight; PY, plot yield; SPAD1 and SPAD2, chlorophyll content at the beginning of the stress treatment and about 4 weeks after onset of drought stress, respectively. PRO, free proline content; TSS, total content of soluble sugars. Bonferroni threshold (-log10 (p) > 5.87), is represented by a continuous grey line. X-axis represents the six faba bean chromosomes. The biggest metacentric chromosome I is divided in two corresponding to the large (L) and short (S) arms. Chromosome 0 stands for unknown locations.

**Figure 6:**
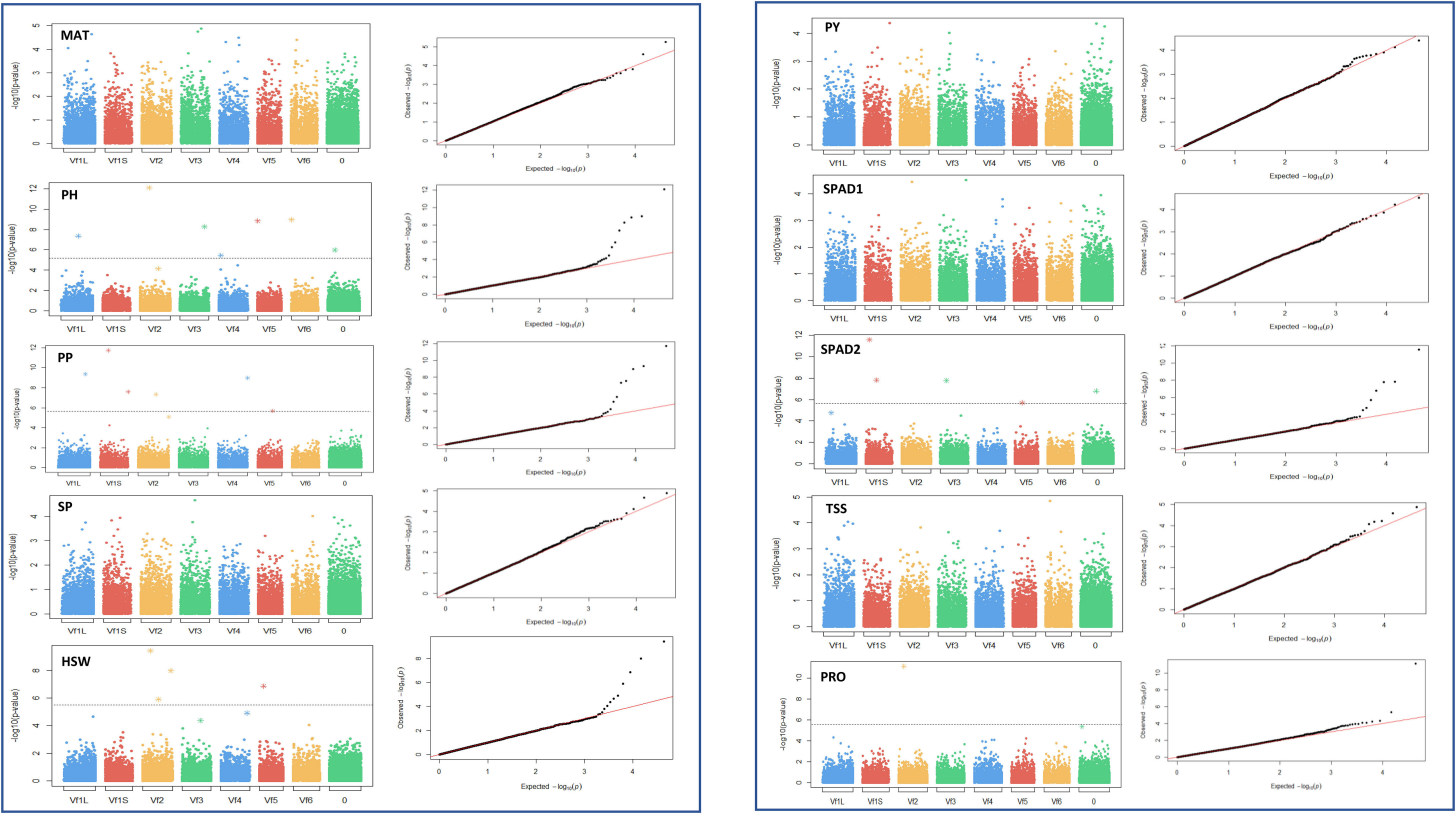
Manhattan plots and quantile-quantile (Q-Q) plots of the GWAS results for the 10 traits studied in drought stress conditions. MAT, maturity date; PH, plant height; PP, number of pods per plant; SP, number of seeds per plant; HSW, 100 seed weight; PY, plot yield; SPAD1 and SPAD2, chlorophyll content at the beginning of the stress treatment and about 4 weeks after onset of drought stress, respectively. PRO, free proline content; TSS, total content of soluble sugars. Bonferroni threshold (-log10 (p) > 5.87), is represented by a continuous grey line. X-axis represents the six faba bean chromosomes. The biggest metacentric chromosome I is divided in two corresponding to the large (L) and short (S) arms. Chromosome 0 stands for unknown locations.

To investigate genetic variants governing drought tolerance in faba bean, 10 morphological, phenological and physiological traits (MAT, PH, PP, SP, HSW, PY, SPAD1, SPAD2, PRO and TSS) were subjected to GWAS analysis using 21,915 SNPs. A total of 74 marker trait associations (MTAs) were identified, revealing candidate loci for each trait across different water regimes (**Tables 2** and **3**). The manhattan and their corresponding quantile-quantile (Q-Q) plots run with the MLMM method are shown in **Figures 5** and **6**. Q-Q plots revealed that the -log10 (p-values) for the different traits evaluated under each water regime condition conformed to normal distribution.

**Table 2:** List of candidate genes related to control assay traits.

Trait^a^	Axiom_Vfaba ID^b^	%R2	Chrom_Vf	MT_Ortholog	Gene Annotation	Mt locations^c^
**PH**	AX-416733403	20.1	VF1L	LOC11420042 - MTR_2g034600	Transcription termination factor MTERF5, chloroplastic	Chr2: 13,305,552 - 13,309,126
	AX-416779256	18.2	VF3	LOC25484925 - MTR_1g094180	Uncharacterized LOC25484925	Chr1: 46,026,865 - 46,030,860
	AX-181204108	8.5	VF2	LOC11434242 - MTR_3g101290	Transcription factor MYB73	Chr3: 50,051,392 - 50,052,915
	AX-416746074	6.9	VF3	LOC25501550 - MTR_8g071050	Uncharacterized LOC25501550	Chr8: 32,595,784 - 32,600,071
	AX-416824745	4.7	VF2	LOC11418507 - MTR_3g093440	SET and MYND domain-containing protein 4	Chr3: 46,160,188 - 46,169,983
	AX-416725335	4.5	VF3	LOC11409185 - MTR_1g017770	U-box domain-containing protein 30	Chr1: 5,096,589 - 5,098,303
	AX-181165425	4.1	VF4	LOC25493784 - MTR_4g107280	Serine/threonine-protein kinase AtPK2/AtPK19	Chr4: 52,305,859 - 52,309, 642
	AX-181460581	3.5	VF5	LOC11429781 - MTR_7g113830	Transcription factor bHLH96	Chr7: 53,954,046 - 53,955,883
**PP**	*AX-416730999*	22.1	VF2	–	No significant similarity found	–
	**AX-416814849**	15.3	VF1S	LOC11411992-MTR_2g081930	Organic cation/carnitine transporter 4	Chr2: 40,519,275 - 40,525,105
	AX-416811873	9.8	VF2	–	No significant similarity found	–
	AX-416736864	9.6	VF3	LOC11433286 - MTR_1g072570	Protein HUA2-LIKE 3	Chr1: 36,125,447 - 36,149,302
	AX-416742185	8.0	VF2	LOC11418689 - MTR_4g007220	Probable fucosyltransferase 7	Chr4: 1,030,501 - 1,033,327
	AX-181168375	0.1	VF5	LOC11436945 - MTR_7g078070	Bifunctional L-3-cyanoalanine synthase/cysteine synthase 1, mitochondrial	Chr7: 36,224,960 - 36,229,173
	AX-416733741	0.1	VF1L	LOC11436596 - MTR_5g090550	TLC domain-containing protein 4	Chr5: 40,621,005 - 40,626,857
	AX-416746789	-0.1	VF2	LOC11406652 - MTR_3g065480	Uncharacterized LOC11406652	Chr3: 33,106,129 - 33,112,502
**SP**	*AX-416730999*	37.2	VF2	–	No significant similarity found	–
	AX-181196762	13.8	VF1L	LOC11407110 - MTR_5g072620	Phenylacetaldehyde reductase	Chr5: 32,215,050 - 32,219,289
	AX-416803016	12.0	VF2	LOC11417256 - MTR_3g073530	RHOMBOID-like protein 8	Chr3: 36,651,502 - 36,655,433
	AX-181481926	10.0	VF1S	LOC25495903 - MTR_6g033275	Uncharacterized LOC25495903	Chr6: 11,097,750 - 11,104,911
	AX-416766053	6.4	VF3	LOC11424057 - MTR_1g056550	Syntaxin-121	Chr1: 28,618,423 - 28,622,050
	AX-416809778	5.1	VF2	LOC25487330 - MTR_2g079050	Proline-rich extensin-like protein EPR1	Chr2: 39,201,706 - 39,205,770
**HSW**	**AX-181155165**	21.3	VF2	LOC11414533 - MTR_4g014600	Protein TIC 56, chloroplastic	Chr4: 4,407,419 - 4,414,567
	**AX-181178618**	17.3	VF2	LOC11423990 - MTR_3g096160	BTB/POZ domain-containing protein At2g13690	Chr3: 47,404,439 - 47,407,638
	AX-416734460	8.9	VF1S	LOC11433077 - MTR_2g103380	Uncharacterized protein At1g15400	Chr2: 50,770,172 - 50,771,135
	AX-416741157	2.3	VF1L	LOC11430458 - MTR_5g083560	Cation/H(+) antiporter 14	Chr5: 37,260,367 - 37,261,590
	AX-181483294	1.9	VF5	LOC11435913 - MTR_7g086510	Cationic amino acid transporter 4, vacuolar	Chr7: 40,354,298 - 40,361,570
	AX-416771643	0.9	VF1L	LOC11426550 - MTR_5g082490	Protein yippee-like	Chr5: 36,649,198 - 36,652,967
	AX-416761080	0.8	VF6	LOC25492837 - MTR_4g074390	Pentatricopeptide repeat-containing protein At1g09820	Chr4: 36,315,927 - 36,318,984
**PY**	AX-181486157	29.2	VF2	LOC11438062 - MTR_3g062440	Pathogenesis-related genes transcriptional activator PTI6	Chr3: 31,638,312 - 31, 639,244
	AX-416726542	12.3	VF4	LOC25493803 - MTR_4g107500	ATPase 11, plasma membrane-type	Chr4: 52,517,283 - 52,523,566
	AX-181457993	12.0	VF6	LOC11438539 - MTR_8g093850	Uncharacterized LOC11438539	Chr8
	AX-416803843	9.6	VF1L	LOC11439965 - MTR_5g008680	Peptidyl-prolyl cis-trans isomerase FKBP53	Chr5: 1,881,201 - 1,886,862
	AX-416794085	8.9	VF1S	LOC25479984 - MTR_0049s0070	Protein NSP-INTERACTING KINASE 2	MtrunA 17r5.0-ANR-Scaffold
	AX-181496354	8.7	VF4	LOC25483593 - MTR_1g054710	rRNA biogenesis protein RRP5	Chr1: 27,658,013 - 27,684,808
	AX-416740528	3.4	VF3	LOC25484291 - MTR_1g070140	RING-H2 finger protein ATL16	Chr1: 34,725,951 - 34,727,735
	AX-181155156	-0.2	VF4	LOC11438091 - MTR_4g108270	Probable serine/threonine-protein kinase PBL3	Chr4: 52,898,547 - 52,903,260
**SPAD1**	AX-416773777	15.3	VF1S	LOC11406053 - MTR_2g086780	OVARIAN TUMOR DOMAIN-containing deubiquitinating enzyme 6	Chr2: 42,569,722 - 42,577,518
	AX-416813816	12.5	VF3	LOC25483937 - MTR_1g062760	Ras-related protein RABC2a, mRNA	Chr1: 31,429,552 - 31,432,831
	AX-416806007	10.4	VF1S	LOC25495703 - MTR_6g022710	Probable carboxylesterase 11	Chr1: 8,147,008 - 8,153,086
	AX-416770296	9.4	VF5	LOC11434746 - MTR_7g10068	Probable DNA helicase MCM8	Chr7: 47,402,118 - 47,411,018
	AX-181469161	8.7	VF6	LOC11415976 - MTR_4g068190	Peptidyl-prolyl cis-trans isomerase FKBP16-3, chloroplastic, mRNA	Chr4: 33,706,200 - 33,709,953
	AX-181162616	3.3	VF4	LOC11408450 - MTR_8g058330	Protein transport protein Sec61 subunit alpha	Chr8: 23,516,578 - 23,520,693
	AX-181157586	3.4	VF2	LOC11438520 - MTR_3g085280	Uncharacterized LOC11438520	Chr3: 41,983,181 - 41,987,055
	AX-416766853	2.2	VF4	LOC11439881 - MTR_8g035560	Receptor-like serine/threonine-protein kinase At2g45590	Chr8: 13,391,605 - 13,394,516
**PRO**	AX-416786927	22.3	VF5	LOC11423151 - MTR_7g112460	Topless-related protein 3	Chr7: 53,265,254 - 53,275,452
	AX-181473167	21.7	VF5	LOC11433065 - MTR_7g104890	Signal recognition particle 54 kDa protein, chloroplastic	Chr7: 49,538,278 - 49,547,049
	AX-181187546	17.7	VF5	LOC11435620 - MTR_7g086430	Pobable zinc metalloprotease EGY1, chloroplastic	Chr7: 40,311,949 - 40,318,392
	AX-181153857	9.5	VF1L	LOC11415575 - MTR_5g070860	AT2G18410-like protein mRNA, partial cds	Chr5: 31,253,236 - 31-258,745
	AX-416734875	3.9	VF1L	LOC11429004 - MTR_5g013970	Phosphoacetylglucosamine mutase, transcript variant X2, mRNA	Chr5: 4,614,512 - 4,620,938
	AX-416766779	0.9	VF4	LOC11420237 - MTR_4g119900	Probable histone-arginine methyltransferase 1.3	Chr4: 57,819,179 - 57,827,376
	AX-181173312	0.5	VF2	LOC25489275 - MTR_3g067650	Probable serine/threonine-protein kinase PBL7	Chr3: 33,731,346 - 33,735,869

ID of the associated single nucleotide polymorphisms (SNPs) in the Vicia faba Axiom, percentage of phenotypic variation explained (%R2), location in the faba bean chromosomes and orthologous genes, annotation and location in Medicago.

(^a^): Plant height (PH), number of pods per plant (PP), number of seeds per plant (SP), 100 seed weight (HSW), plot yield (PY), chlorophyll content (SPAD1), free proline content (PRO), and total content of soluble sugars (TSS).

(^b^): In bold, loci associated with HSW, SPAD1 and PRO in both conditions. In red, significant loci associated to the traits that did not reach the Bonferroni threshold (p) > 5.83. In italics, common loci associated with PP and SP traits.

(c): Gene locations were determined using the Genome Data Viewer (GDV).

**Table 3:** List of candidate genes related to drought resistance traits.

Trait^a^	Axiom_Vfaba ID^b^	%R2	Chrom_Vf	MT_Ortholog	Gene Annotation	Mt locations^c^
**PH**	AX-181182113	15.4	VF5	LOC11435290 - MTR_2g104100	Formamidopyrimidine-DNA glycosylase	Chr2: 51,106,899 - 51,114,875
	AX-416815601	14.9	VF2	LOC11431814 - MTR_3g101520	B3 domain-containing protein At3g19184	Chr3: 50,178,330 - 50,183,223
	AX-416734003	14.6	VF2	LOC25489500 - MTR_3g075100	Telomere length regulation protein TEL2 homolog	Chr3: 37,598,521 - 37,609,730
	AX-181183872	11.7	VF4	LOC25501139 - MTR_8g044260	DExH-box ATP-dependent RNA helicase DExH3	Chr8:16,827,348 - 16,850,409
	AX-181483303	8.4	VF0	LOC11434981 - MTR_1g045510	28 kDa ribonucleoprotein, chloroplastic	Chr1: 17,459,568 - 17,462,374
	AX-181167806	5.9	VF6	LOC25494322 - MTR_4g133952	Nuclear transcription factor Y subunit B-3	Chr4:64,266,289 - 64,269,332
	AX-416758364	4.9	VF3	LOC11432831 - MTR_1g009620	Exocyst complex component EXO70I	Chr1: 1,426,882 - 1,430,478
	AX-181481274	2.8	VF1L	LOC11408589 - MTR_5g024350	Glutamate receptor 3.6	Chr5: 9,819,043 - 9,825,020
**PP**	**AX-416814849**	20.6	VF1S	LOC11411992 - MTR_2g081930	Organic cation/carnitine transporter 4	Chr2: 40,519,275 - 40,525,105
	AX-416774842	12.4	VF4	LOC11445678 - MTR_8g022980	AT-rich interactive domain-containing protein 4	Chr8: 8,135,620 - 8,147,219
	AX-416785709	12.0	VF2	–	No significant similarity found	–
	AX-416750038	11.6	VF1L	LOC25486287- MTR_2g029340	Protein ROOT HAIR DEFECTIVE 3 homolog 2	Chr2:11,013,670 - 11,027,992
	AX-416815627	7.1	VF1S	LOC25496020 - MTR_6g034195	Plastidial pyruvate kinase 2	Chr6: 12,142,869 - 12,151,041
	AX-416817671	3.2	VF5	LOC25499196 - MTR_7g096080	Tryptophan synthase alpha chain	Chr7: 45,376,885 - 45,380,449
	AX-181486430	2.7	VF2	LOC25491358 - MTR_4g011600	Molybdate transporter 2	Chr4: 3,007,294 - 3,009,858
**HSW**	**AX-181155165**	20.3	VF2	LOC11414533 - MTR_4g014600	Protein TIC 56, chloroplastic	Chr4: 4,407,419 - 4,414,567
	AX-416774496	14.5	VF2	LOC11422606 - MTR_3g070390	Nuclear pore complex protein NUP88	Chr3: 35,050,851 - 35,063,461
	**AX-181178618**	13.4	VF2	LOC11423990 - MTR_3g096160	BTB/POZ domain-containing protein At2g13690	Chr3: 47,404,439 - 47,407,638
	AX-181484267	9.7	VF5	LOC11431692 - MTR_7g052640	Putative lipid-transfer protein DIR1	Chr7: 24,973,213 - 24,973,970
	AX-416801263	6.5	VF3	LOC25495073 - MTR_6g003960	Transcription termination factor MTEF1	Chr6: 32,604 - 41,403
	AX-416740666	3.9	VF4	LOC11446464 - MTR_7g093530	Probable xyloglucan endotransglucosylase/hydrolase protein 23	Chr7: 44,048,945 - 44,050,479
**SPAD2**	AX-416771580	26.0	VF1S	LOC11422670 - MTR_2g097800	Uncharacterized LOC11422670	Chr2: 47,890,406 - 47,896,247
	AX-416751689	20.4	VF3	LOC25484157 - MTR_1g069165	CLIP-associated protein	Chr1: 33,665,872 - 33,684,619
	AX-416739735	11.8	VF0	LOC11412171 - MTR_5g021260	Probable ubiquitin-conjugating enzyme E2 16	Chr5: 8,210,511 - 8,216,281
	AX-416800399	10.0	VF1S	LOC11415177 - MTR_6g065110	Probable carboxylesterase 18	Chr1: 31,948,768 - 31,950,002
	AX-416757391	4.0	VF5	LOC11421925 - MTR_1g115950	Uncharacterized LOC11421925	Chr1: 56,096,020 - 56,103,099
	AX-181178687	3.2	VF1L	LOC11411195 - MTR_5g080880	Uncharacterized protein At2g39795, mitochondrial	Chr5: 35,837,184 - 35,842,681
**PRO**	AX-416814537	30.1	VF2	LOC25490802 - MTR_3g462820	Beta-glucosidase BoGH3B	Chr3: 28,335,676 - 28,340,509,
	AX-181191699	13.0	VF2	LOC11433803 - MTR_3g089510	Mitogen-activated protein kinase 20	Chr3: 20,834,197 - 20, 842,245

ID of the associated single nucleotide polymorphisms (SNPs) in the *Vicia faba* Axiom, percentage of phenotypic variation explained (%R2), location in the faba bean chromosomes and orthologous genes, annotation and location in Medicago. ^a^)Plant height (PH), number of pods per plant (PP), 100 seed weight (HSW), chlorophyll content (SPAD2), free proline content (PRO).

(^b^)In bold, loci associated with HSW in both conditions. In red, significant loci associated to the traits that did not reach the Bonferroni threshold (p) > 5.83.

(^c^)Gene locations were determined using the Genome Data Viewer (GDV).

Under control conditions we detected 52 significant SNPs spread along the genome, although 17 did not reach the Bonferroni threshold -log10(p) > 5.83. Most of the significant markers, however, clustered in chromosome 1 and 2 (**Table 2**). Eight loci were associated with PH, PP, PY and SPAD1, accounting together for 70.5%, 64.9%, 84% and 65.3% of the respective trait variation. Likewise, seven significant SNPs captured 53.4% of the HSW and 76.4% of the PRO variation. Finally, the six markers associated with SP provided the highest contribution to the phenotypic variance (84.6%). No significant associations were detected for MAT, SPAD2 and TSS. The SNP AX-416730999 has a common association with PP and SP. No such colocalization of SNP markers with multiple traits was observed under drought conditions.

Under drought stress, a total of 29 loci were significantly associated with the traits although 8 did not reach the Bonferroni threshold -log10(p) > 5.83 (**Table 3**). Although distributed across the six faba bean chromosomes, 16 of them (55%) colocalized mostly in chromosomes 1 and 2 while three of them could not be assigned. Six markers were HSW and SPAD2 associated, jointly explaining 68.4% and 75.4% of the trait variation, respectively. Eight SNPs were associated with PH and seven with PP explaining, respectively, 78.6%, 69.6% of the phenotypic variation. No SNPs associated with MAT, SP and PY were found. The GWAS analysis did not identify significant SNP markers for SPAD1 and TSS, but PRO showed association with two SNPs, one of them explaining the highest percentages of the trait variation in drought conditions (30.1%). Three pleiotropic loci (noted in bold in **Tables 2** and **3**) were associated with HSW and PP in both water regime conditions.

To further understand the genetic basis of faba bean drought stress-related traits, the sequences flanking SNPs associated with significant traits were subjected to a BLAST search to identify the orthologous sequences in *M. truncatula* or in other model legumes. From the 29 candidate genes (**Table 3**), 28 were functionally annotated while one of them did not show a significant sequence similarity with *Medicago*. For the sake of brevity, we will mainly comment on the putative genes explaining around 10% of the trait variation.

Starting with the phenological and yield related traits, eight significant SNPs were found for plant height (PH). Four of these genes, annotated as formamidopyrimidine-DNA glycosylase, B3 domain-containing protein At3g19184, Telomere length regulation protein TEL2 and DExH-box ATP-dependent RNA helicase DExH3 (**Table 3**), accounted for 15.4%, 14.9%, 14.6% and 11.7%, respectively, of the phenotypic variation. Concerning pods per plant (PP), the most significant gene explaining 20.6% of the variation is annotated as organic cation/carnitine transporter 4. Another putative candidate gene explained 12.4% of the trait variation and corresponds to a AT-rich interactive domain-containing protein 4 and a Protein ROOT HAIR DEFECTIVE 3 homolog 2 accounted for 11.6% of the variation. Finally, one uncharacterized SNP explained 12% of the trait. Six putative candidates were associated with hundred seed weight (HSW). The first of these, Protein TIC 56, which contributes to 20.3% of the variation, participates at the inner chloroplast envelope membrane to form a channel for plastid protein import (Kikuchi et al., 2013). Next, a nuclear pore complex protein NUP88, explained 14.5% of the variation, while another gene annotated as a BTB/POZ domain-containing protein explained 13.4% of the trait.

Concerning the physiological traits, six SNPs were significantly associated with SPAD2 and two of them, the uncharacterized LOC11422670 and a CLIP-associated protein, explained the highest percentage of variation (26% and 20.4%, respectively). Besides, a ubiquitin-conjugating enzyme E2 16 together with a probable carboxylesterase 18 were responsible for 11.8 and 10% of the SPAD2 variation. For proline content (PRO), two SNPs annotated as beta-glucosidase BoGH3B and mitogen-activated protein kinase 20 accounted for a significant percentage of the trait variation (30.1% and 13%, respectively).

## Discussion

Drought stress represents a major threat to plant growth and development. Since faba bean is generally grown under rainfed conditions, it often experiences water stress at the terminal growth phase of the crop. Considering the present scenario of climate change, enhancing faba bean productivity through improved drought tolerance is a prioritary goal in breeding efforts.

Phenotyping for drought tolerance is costly and time consuming, but secondary characters with high heritability that are correlated with yield under drought conditions can be used for indirect selection (Ziyomo and Bernardo, 2013). In the present study, 100 faba bean accessions from different origins were evaluated for two years under control and drought conditions, for 10 secondary traits associated to phenology, physiology and grain yield. These traits have been used in different legume studies for efficient assessment of stress tolerance (Nadeem et al., 2019; Valdisser et al., 2020; Ravelombola et al., 2021; Wu et al., 2021). Considering both years and conditions, the yield related traits (PP, SP, HSW, PY) were highly correlated with MAT, PH and SPAD2 and could thus be used as efficient secondary traits for drought tolerance in faba bean improvement programs. Accordingly, the vectors among these traits in **Figure 4** had small angles confirming the positive correlation. The selection of faba bean accessions from different origins, with sufficient genetic variation and weak population structure revealed a large variation in these traits, suggesting that the panel is genetically diverse and could be advantageous for GWAS implementation.

GWAS has become a critical tool for detecting genetic variants underlying complex traits. The large number of SNPs obtained with the 50K SNP array from Affymetrix (O’Sullivan et al., 2019; Khazaei et al., 2021) has provided an extensive genome coverage to differentiate germplasm accessions and to carry out high-resolution association mapping. Using 21,915 SNPs we detected here a total of 52 significant SNPs in irrigation (control) and 29 in drought conditions, distributed across the six faba bean chromosomes, which collectively explained a high percentage of the total phenotypic variation. Three of these SNPs were associated with the traits evaluated both under control and drought conditions (**Tables 2** and **3**) and should thus not be relevant to drought stress. In control conditions the SNPs associated with morphological, physiological and yield related traits explained from 53.4% in the case of HSW to 84.6% in SP while under drought stress the R2 values ranged from 43.1% (PRO) to 78.6% (PH). No significantly associated SNPs were detected for MAT, SPAD2 and TSS in control conditions or for MAT, SP, PY, SPAD1 and TSS under stress. Interestingly, two significant SNPs accounting for high percentages of the trait variation in PP and SPAD2, correspond to uncharacterized proteins indicating the presence of novel genes associated with drought tolerance in faba bean.

To progress in our understanding and possible functions of significant genes, we investigated their involvement in water stress-responses reported from other crop species. BLAST search analysis showed that most of the significant SNP markers identified in the present study aligned with candidates, known to be involved in responses to drought stress in different crops (**Table 3**).

Four main drought stress response candidates were identified for PH. The first corresponds to a formamidopyrimidine-DNA glycosylase reported to initiate base excision repair at damaged sites in response to abiotic stresses (Chen et al., 2012; Wallace, 2014). The expression of many drought-induced genes is regulated at the transcriptional level and this activity can be extended to the second candidate, a B3 domain-containing protein At3g19184 candidate since in maize, the B3 domain-containing transcription factor Viviparous1 (Vp1) was induced by drought stress (Cao et al., 2007). The third major candidate identified in our study is a homolog of the telomere length regulation protein TEL2, a key regulator of cell proliferation and genome maintenance. TEL2 complexes interacts with and promotes protein kinases stability by controlling telomerase length as well as the DNA damage response (Smogorzewska and de Lange, 2004; Smogorzewska and de Lange, 2004; Sugimoto, 2018). For example, a telomere length regulation protein TEL2 homolog in rice was differentially expressed in response to salinity stress (Cotsaftis et al., 2011). Finally, the fourth candidate gene encodes a DExH-box ATP-dependent RNA helicase, which in plants has a critical role in a variety of RNA-mediated regulation of cell proliferation and abiotic stress responses (Liu and Imai, 2018). In addition, four other candidates genes detected in our work encode, respectively, a 28 kDa ribonucleoprotein, a which was recently reported in chickpea response to biotic and abiotic stresses (Vessal et al., 2020); a nuclear Y-B transcription factor that has proven to regulate resistance to drought stress in *Arabidopsis*, maize and soybean (Nelson et al., 2007; Sun et al., 2022); an exocyst complex component reported as a drought and salt tolerance regulator in grapevine (Wang et al., 2023), and a glutamate receptor with a signalling role in responses to abiotic stresses such as salt, cold, heat, and drought, of Arabidopsis, faba bean and rice (Lu et al., 2014; Yoshida et al., 2016; Qiu et al., 2019).

Concerning the number of pods per plant (PP), an organic cation/carnitine transporter 4, an AT-rich interactive domain-containing protein (ARID domain) and a protein ROOT HAIR DEFECTIVE 3 (RHD3) homolog 2 were the most significant annotated genes identified. In *Arabidopsis*, several organic cation transporters were up-regulated during drought stress suggesting a specific role in plant adaptation to environmental stress (Küfner and Koch, 2008). Likewise, the ARID domain containing proteins are transcription factors implicated in a wide variety of roles, including chromatin remodelling, transcription, and cell growth (Wilsker et al., 2002). In a proteomics study of sugarcane response, (Salvato et al., 2019) showed that different types of transcriptional regulators, including ARID domains proteins were differentially accumulated in response to drought stress. RHD3 was also required for regulation of cell expansion and root hair development. Thus, Wong et al. (2018) reported that the ectopic expression of a *Musa acuminata* RHD3 gene enhanced drought tolerance in *Arabidopsis*. Moreover, a root transcriptomic analysis of contrasting *Gossypium herbaceum* genotypes revealed a higher expression of RHD3 genes in tolerant lines, highlighting the key involvement of these genes in root length development and plasticity under drought stress conditions (Ranjan et al., 2012).

Three main QTLs were associated with hundred seed weight (HSW): Protein TIC 56 participates at the inner chloroplast envelope membrane to form a channel for plastid protein import (Kikuchi et al., 2013), a BTB/POZ domain-containing protein with potential roles in developmental programs such as promotion of leaf and floral meristem fate and determinacy, as well as in defence and abiotic stress response (Guan et al., 2018). Both genes (Protein TIC 56 and BTB/POZ domain) were significant both under control and drought conditions. The next, candidate is the nuclear pore complex (NPC) protein NUP88. Diverse mechanisms have been proposed to explain the role of NPC family components in responses to different stresses such as cold, abscisic acid (ABA), drought, and biotic stress (Yang et al., 2017). Further candidates associated with HSW in drought conditions include the lipid-transfer protein (LTP) DIR1, the transcription termination factor MTEF1 and a xyloglucan endotransglucosylase/hydrolase protein. LTPs are thought to be involved in plant defense responses (Safi et al., 2015) and their expression is induced by biotic and abiotic stresses, including disease, salinity, temperature and drought (Safi et al., 2015; Akhiyarova et al., 2021; Duo et al., 2021; Zhao et al., 2021). It is also well established that drought tolerance is regulated by the mitochondrial transcription termination factors (MTERFs). A recent analysis of *mterf* mutants supports aa role for plant MTERFs in abiotic stress response (Quesada, 2016). Similarly, the xyloglucan endotransglucosylase/hydrolases are inducible by a broad spectrum of abiotic stresses and have been shown to enhanced tolerance to salt and drought stresses in tomato (Choi et al., 2011).

Concerning the physiological trait SPAD2, only three of the six significant SNPs were annotated, indicating the presence of three novel candidates associated with drought tolerance in faba bean. The first of the annotated candidates, a CLIP-associated protein (CLASPs), correspond to an evolutionarily conserved family of regulatory factors that control microtubule dynamics and the organization of microtubule networks. Although little is known about their function in plants, in Arabidopsis, CLASP is involved in both cell division and cell expansion by linking microtubules and auxin transport (Ambrose et al., 2007). The second candidate gene encodes an ubiquitin-conjugating enzyme that has shown to enhance drought and salt tolerance in *Arabidopsis* and melon, as well as in different legume crops such as soybean, peanut or mung bean (Zhou et al., 2010; Baloglu and Patir, 2014; Wang et al., 2016; Chen et al., 2020). Finally, carboxylesterases are known to play important roles in plant growth, development and resistance to stresses (Prinsi et al., 2018; Arisha et al., 2020; Rui et al., 2022).

The two candidates related to proline content (PRO) were the beta-glucosidase BoGH3B and the mitogen-activated protein kinase 20. Plant β-glucosidases are involved in cell wall biogenesis which protects plants against external stresses (Moradi Tarnabi et al., 2020). Different b-glucosidase homologs were shown to be involved in the response to dehydration and NaCl stress in *Arabidopsis* (Xu et al., 2012) and drought stress in soybean roots (Wang et al., 2017). Finally, mitogen-activated protein kinase (MAPK) genes are involved in many cell activities including growth, differentiation and proliferation, as well as environmental stress responses. MAPKs activation is a common defense response of plants to a range of abiotic stressors (Komis et al., 2018; Muhammad et al., 2019; Kim et al., 2021). Because drought stress leads simultaneously to osmotic and oxidative stress (Zhu, 2002), osmotic stress activates several protein kinases including MAPKs, which mediate osmotic homeostasis and/or detoxification responses.

Osmotic adaptation is a major component of drought resistance in different crops (Sánchez et al., 1998; Bajji et al., 2001). Proline and soluble sugar accumulation are common physiological responses in many plants during water-deficit stress, to protect cellular components and to restore the osmotic balance (Chen and Murata, 2002; Guo et al., 2018). Accordingly, PRO and TSS increased under drought stress (**Table 1**). Severe drought stress also inhibits the photosynthesis of plants by causing a decrease in chlorophyll content (Ommen et al., 1999). Our results reveal that four weeks after the onset of stress, the mean chlorophyll content (SPAD2) was highly reduced mainly due to damage in chloroplasts caused by reactive oxygen species (Smirnoff, 1995).

The wide range of candidates functionally annotated and significantly associated with drought stress component traits evidences that drought responses are complex and that each induction phase may be controlled by different signalling mechanisms and transcription factors (Shinozaki et al., 2003) classified the products of stress-inducible genes identified in microarray experiments into two groups, one includes molecules such as late embryogenesis abundant (LEA) proteins, osmotin, key enzymes for osmolyte biosynthesis, water channel proteins, sugar and proline transporters, and various proteases, while the second group consists of regulators of intracellular signalling and stress-inducible gene expression (e.g. protein kinases such as MAP kinases, phosphatases, phospholipid metabolic enzymes, and various types of transcription factors). A mitogen-activated protein kinase and a plastidial pyruvate kinase were associated with PRO and PP respectively, while two transcription factors were significantly associated with PP and HSW. Receptor kinases are considered as key regulators of plant architecture and growth, but they also function in defence and stress responses (Marshall et al., 2012). In fact, some serine/threonine-protein kinases are known to play a role in signal transduction and were shown to improve drought tolerance in Arabidopsis, rice, soja and bamboo (Xie et al., 2014; Liu et al., 2022). On the other hand, it is well known that transcription factors synchronise signal transduction and expression of drought tolerance regulatory genes, enabling plants to withstand stress conditions (Joshi et al., 2016; Hrmova and Hussain, 2021). For these reasons, they are considered as potential candidates with broad applications in crop breeding. These results show that the approach applied to this faba bean collection could lead to the efficient identification of candidate genes that are relevant to faba bean drought tolerance.

In summary, our study demonstrates the feasibility of GWAS analysis with a diverse germplasm collection and a high-density array chip, for the identification of drought tolerance-related traits in faba bean. Under stress conditions, 29 SNP markers that were significantly correlated to these traits have been identified, mostly clustered in chromosomes 1 and 2. Interestingly, all of them were directly or indirectly involved in responses to drought stress, thus establishing a solid foundation for further research. The identification of a number of stress-inducible signal transducers provides valuable information on the putative faba bean response mechanisms against drought stress. Nevertheless, a validation of the identified markers in a larger size or bi-parental population, using tissue and stage specific gene expression data from RNA-Seq, would be reasonable before embarking on a broad breeding program. The results from this study will contribute to a better understanding of the genetic architecture governing drought tolerance in faba bean and provide a foundation for marker-assisted breeding in this crop.

## Data availability statement

The original contributions presented in the study are included in the article/**Supplementary Material**. Further inquiries can be directed to the corresponding author.

## Author contributions

AT and NG selected and multiplied the seeds for the experiments and devised the project. CB planned and carried out the drought evaluations and measurements. MP designed and supervised the statistical analysis. NG processed the experimental data and performed the GWAS analysis. AT and NG wrote the manuscript in consultation with MP and CB. All authors contributed to the article and approved the submitted version.

## Footnotes

https://projects.au.dk/fabagenome/genomics-data

2
https://www.geneious.com

3
www.blast.ncbi.nlm.nih.gov/Blast.cgi

4
www.ncbi.nlm.nih.gov/genome/gdv/
