# Supplementary figures and images for: Genome-wide association analysis for drought tolerance and associated traits in faba bean (Vicia faba L.)
Source: Front Plant Sci. 2023 Feb 1;14:1091875. doi: 10.3389/fpls.2023.1091875 (PMC9928957; doi:10.3389/fpls.2023.1091875)

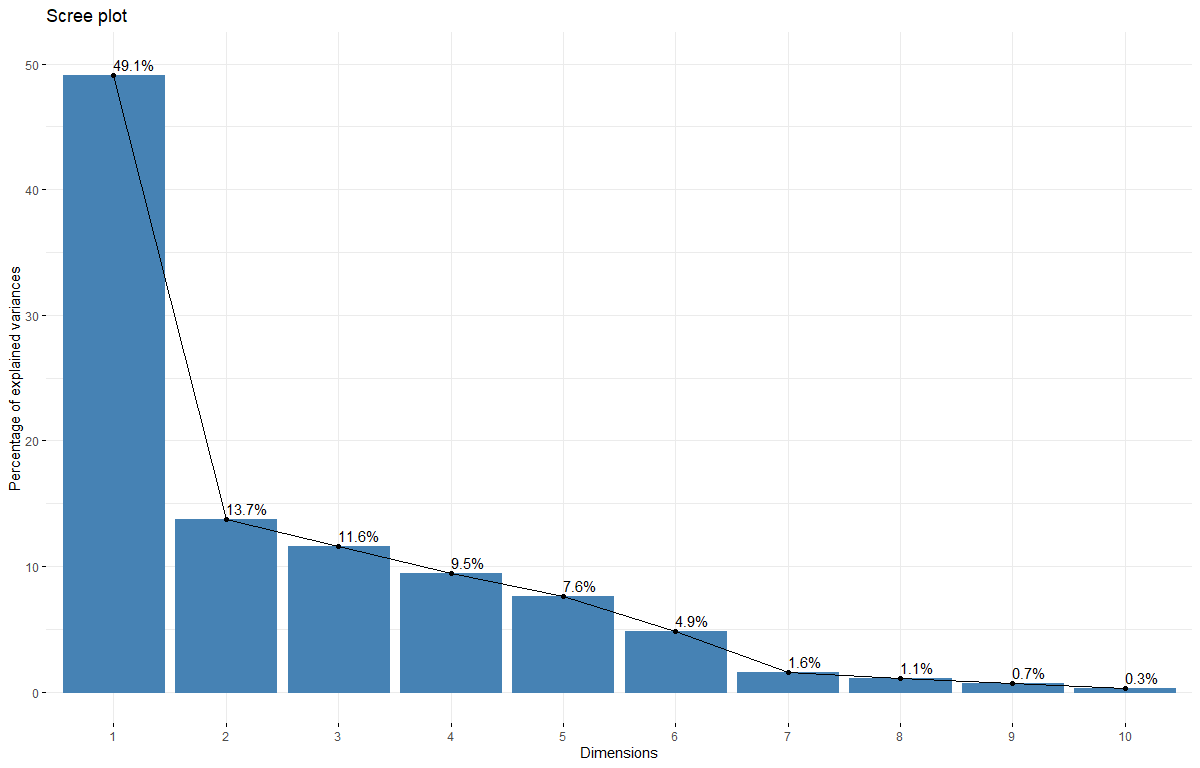

Supplement: Supplementary file 1 [file Image_1.jpeg]

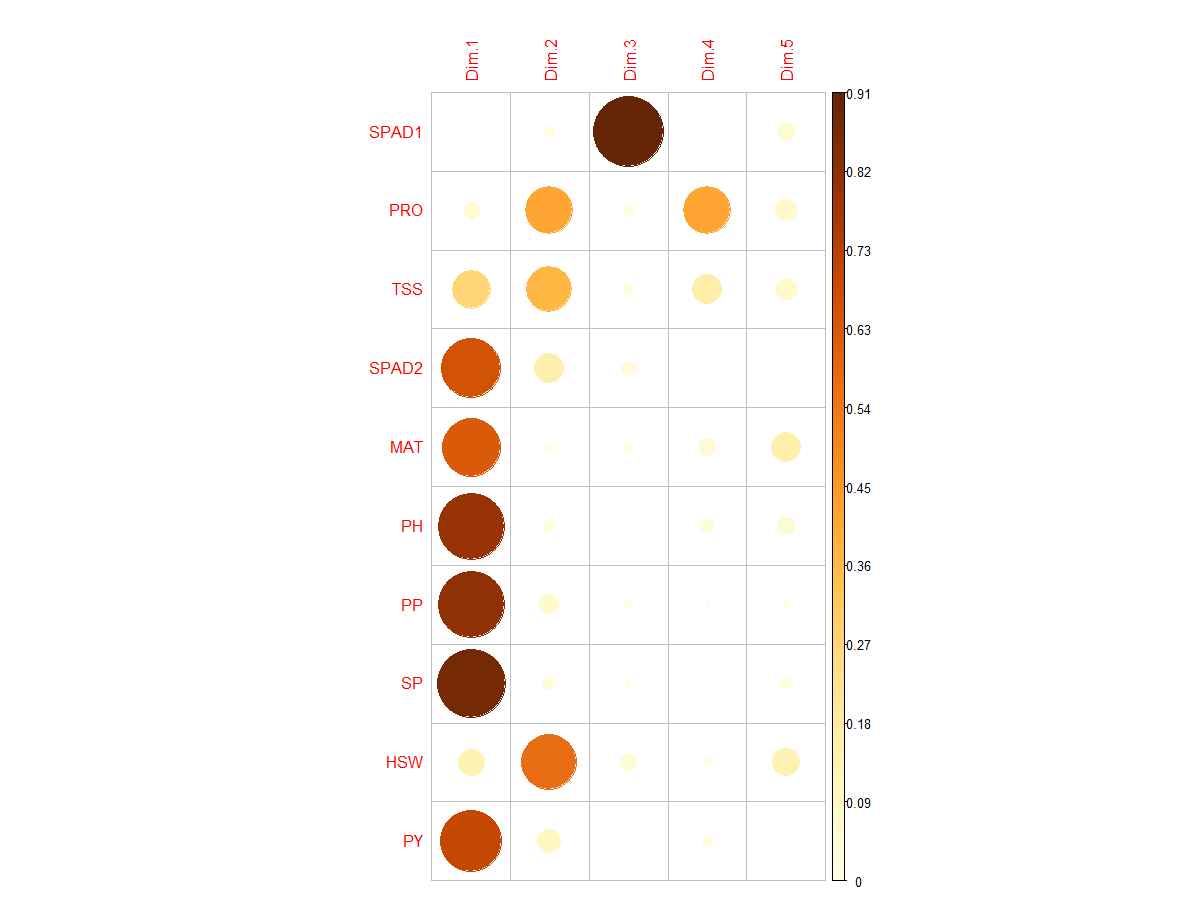

Supplement: Supplementary file 2 [file Image_2.jpeg]
